# Environmental uncertainty and digital technologies corporate in shaping corporate green behavior and tax avoidance

**DOI:** 10.1038/s41598-023-49687-w

**Published:** 2023-12-13

**Authors:** Xiang-Yuan Ao, Tze San Ong, Roberto Aprile, Assunta Di Vaio

**Affiliations:** 1 School of Business and Economics, Universiti Putra Malaysia, Serdang, Malaysia; 2 Department of Business Administration, Daffodil International University, Dhaka, Bangladesh; 3 Department of Management, University of Bergamo, Bergamo, Italy; 4 Department of Law, University of Naples “Parthenope”, Naples, Italy; 5 Present Address: School of Management, Guangzhou University, Guangdong , China

**Keywords:** Environmental sciences, Sustainability

## Abstract

This study contributes to the field of sustainability by analyzing changes in firms following the adoption of new environmental protection laws to meet community sustainability needs. Focusing on the Chinese context, it examined the relationship between firms' environmental protection measures (i.e., corporate green behavior) and profitability (i.e., corporate tax avoidance). The moderating roles of environmental uncertainty and digital technology application in this relationship were also investigated. The findings offer insights into the complex dynamics linking firms’ environmental initiatives to their business outcomes and financial decisions within the framework of a sustainable community. Ultimately, this study highlights the importance and implications of sustainable practices for both the environment and corporate financial performance. Firms’ environmental behaviors are enablers of sustainable communities by deploying natural resources and creating a more resilient economy through active community participation in green production models.

## Introduction

The triple planetary crises of climate change, biodiversity loss, and pollution can be attributed to the underlying unsustainable patterns of consumption and production, posing a significant threat to both living beings and the achievement of the Sustainable Development Goals (SDGs) outlined by the United Nations (UN). China is currently grappling with the impact of this crisis, which is a result of its rapid economic expansion through aggressive urbanization and industrialization^1^. The Chinese government has therefore taken a proactive approach towards achieving the SDGs^2^, on the basis that government and stakeholder collaboration is essential for higher resource efficiency, waste and pollution reduction, and circular economy formation^3^. Accordingly, Miras-Rodriguez et al.^4^ reported that China has pledged to reach peak carbon emissions by the year 2030 through the decrease of carbon intensity by 60%. Moreover, the integration of energy conservation measures into China's sustainable development strategy is emphasized in the nation’s three most recent five-year plans, as documented by Gao et al.^5^ and Hu et al.^6^

The full implementation of the country’s Environmental Protection Law in 2015 has increased environmental regulation costs and environmental governance pressure for corporations. Consequently, Chinese firms are embracing green innovations in their production processes^7^ by increasing investment and developing innovative methods^8^. Against this backdrop, this study centered on the Chinese context for several compelling reasons. The urgency and magnitude of environmental challenges stemming from rapid economic expansion in China are of paramount concern. As per the World Trade Organization (WTO)^9^, there is a growing imperative to align China's objectives with international sustainability goals. Additionally, China has been actively developing a regulatory framework that promotes sustainable practices, offering economic incentives to encourage green investments. Moreover, the global repercussions of China's actions on environmental and sustainability matters cannot be overlooked, making it a pivotal focal point for research and analysis^2,8,10^. Focusing further on this study’s contenxt, existing literature has examined the individual relationships between corporate green behavior (CGB), corporate tax avoidance (CTA), environmental uncertainty (EU), and digital technology application (DTA); however, few studies have integrated these factors from the perspective of SDG 11 (sustainable cities and communities) and SDG 12 (ensuring sustainable consumption and production patterns). Specifically, there is a need to investigate how EU, characterized by unpredictable environmental factors^11^, influences the association between CGB and CTA. Additionally, the role of DTA, such as smart city solutions, internet of things (IoT), and data analytics, in moderating the link between CGB and CTA requires further exploration. These research gaps present an opportunity to bridge the knowledge divide and offer insights that are crucial for policymakers, businesses, and stakeholders to develop effective sustainable strategies and initiatives. By addressing these gaps, this study contributes to the development of evidence-based practices that foster environmental sustainability and fiscal responsibility in the pursuit of SDGs 11 and 12.

## Theoretical background

According to Philips^12^, stakeholder theory is a management- and ethics-based framework that emphasizes the importance of considering the interests and concerns of various stakeholders (individuals, groups, or entities) when making decisions and conducting business operations. Ali et al.^13^ explained that in the context of sustainability, stakeholder theory underscores the significance of incorporating environmental, social, and governance (ESG) considerations into corporate strategies and practices to achieve long-term sustainable outcomes. It thus plays a significant role in shaping the sustainability framework, especially in the context of the SDGs and the promotion of CGB^14^. By delving into the idea that businesses have a moral and ethical responsibility to consider the interests of all stakeholders, this theory has been influential in shaping sustainability practices, including CGB. In this study, stakeholder theory is used to acknowledge that firms often adjust their behavior in response to stakeholder influences. As stakeholders demand more CGB, organizations are motivated to adopt sustainable practices to maintain positive stakeholder relationships^3,6^.

The flexible allocation and complementarity of firms’ resources are critical in addressing the challenges and uncertainties associated with sustainability initiatives. In this regard, the adoption of the resource-based theory (RBT) in this study elucidates the association between increasingly severe environmental conditions and a progressive economy^15^. The theory posits that firms possess diverse tangible and intangible resources, which can be transformed into unique competencies that cannot be replicated by competitors^16^. According to Ali et al.^13^, the SDGs provide a comprehensive roadmap for global sustainability, which encompasses environmental, social, and economic dimensions. To achieve the SDGs across all these dimensions, the RBT suggests that organizations, including businesses and governments, should strategically leverage their unique resources and capabilities to plan policies, develop new business models, and implement strategies that align with sustainability goals.

Additionally, the theory of competitive strategy supports this investigation because it provides an explanation for "competitive heterogeneity," or the variation in company performance and the diversity of factors that contribute to that variation^15,17^. Prior studies have had various views about corporate environmental strategy, especially on the relationship between environmental management and corporate financial performance. Scholars generally believe that environmental strategy is beneficial for value creation^18,19^; for instance, Clarkson et al.^20^ found that environmental activities improve financial performance^21^, while Farza et al.^8^ revealed a significant positive correlation between environmental performance and financial indicators like return on equity and return on assets.

According to institutional theory, organizations face common institutional pressures that lead them to take similar strategic activities to gain legitimacy^22^ and social acceptance^23–25^. Following this logic, governmental laws and regulations exert coercive pressure on firms by demanding conformity^26^. In China, strict law enforcement calls for organizations to improve sustainable practices and green innovations^27^ for sustainable communities, to satisfy not only SDG 11 but also government demands. In addition, normative pressures within a sector might push businesses to go above and beyond in their environmental management practices^28^. Corporations are under normative pressure from various industries and subsets of society to abide by a set of norms and procedures to be considered legitimate^29^. As mentioned earlier, the sustainability framework extends beyond the environmental dimension and encompasses social and economic dimensions^13^. In this context, institutional theory illustrates how organizations can navigate the challenges of planning policies, adopting new business models, developing strategies, measuring results, and reducing uncertainty in sustainability implementation. It emphasizes the role of institutions in shaping organizational behavior like CGB, along with the importance of aligning with institutional pressures and norms across all dimensions of sustainability. Therefore, the institutional theory supports this study’s framework by substantiating that firms’ adoption of CGB is motivated by the three institutional pressures (regulatory, mimetic, and normative) imposed by the government and society.

CGB in sustainable communities aligns with the overarching goal of environmental protection and sustainability. By reducing carbon emissions, conserving natural resources, and fostering biodiversity through a range of mitigation measures, firms in these communities demonstrate their commitment to creating a healthier and more sustainable environment for residents and future generations^30^. Specifically, they can mitigate the effects of climate change, improve air quality, and enhance the quality of life for residents^12,31^ while promoting corporate social responsibility and sustainable business practices^32^.

To build a more resilient economy, many governments, including China, offer tax incentives and deductions to businesses that engage in CGB. These incentives are designed to encourage firms to reduce their carbon footprint by adopting eco-friendly practices, investing in renewable energy sources, or improving energy efficiency^32,33^. In this context, CGB contributes to the resilience of sustainable communities by reducing their vulnerability to climate-related risks and resource scarcity^34–36^. Moreover, one of the core objectives of sustainable development is to ensure long-term economic stability and growth^37,38^, which is more likely to be attained in nations with higher economic resilience because they are better prepared to handle environmental, social, and economic challenges. Therefore, businesses that take advantage of the aforementioned tax incentives and prioritize CGB can not only lower their tax liabilities and enhance their economic resilience, but also achieve the benefit of long-term economic, social, and environmental sustainability^39,40^. Reducing their reliance on carbon-intensive operations and energy sources leaves them less vulnerable to potential carbon taxes or regulatory penalties that may be imposed in the future as governments intensify efforts to combat climate change^35,41^.

In addition, DTA fosters innovation and the development of new digital products, including green technologies^30^. For example, the Internet of Things (IoT) can enable smart energy management systems, while artificial intelligence (AI) can optimize transportation routes to reduce emissions. These innovations contribute to CGB by offering sustainable solutions. In this study, digitalization and resilience are closely tied to green behaviors that enable organizations to monitor and optimize their sustainability efforts, enhance supply chain resilience, drive innovation in green technologies, and facilitate transparent sustainability reporting. These digital strategies not only promote CGB but also contribute to the overall resilience of businesses and economies in the face of environmental challenges and disruptions^37,40^.

### Research framework

**Figure 1:**
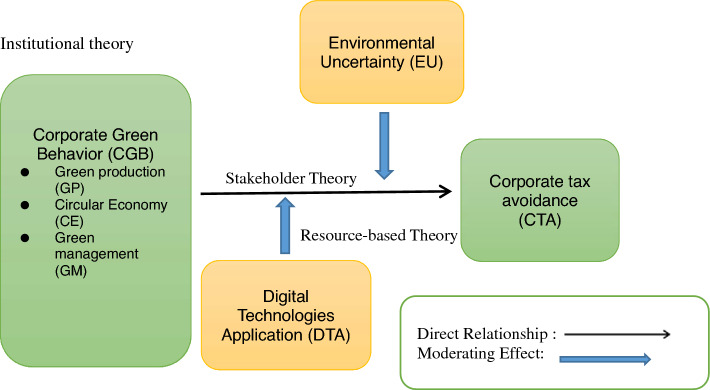
Research framework processed by the authors.

First, from the stakeholder theory viewpoint, firms in China can improve tax avoidance by embracing CGB in alignment with the interests and expectations of various stakeholders. CBG allows firms to access tax incentives, attract investors and customers, maintain positive relationships with communities, and create a more efficient supply chain, all of which contribute to enhanced financial performance and, ultimately, tax efficiency^11,14^. Meanwhile, from the institutional theory perspective, CGB in China is influenced by regulatory, normative, and mimetic pressures. Firms that engage in environmentally sustainable practices can not only comply with regulations and societal norms but also position themselves to take advantage of tax incentives and benefits associated with environmental stewardship. This alignment with institutional pressures can help improve tax avoidance while also contributing to broader environmental goals and corporate social responsibility. Next, the RBT is a framework in strategic management that examines how a firm's unique resources and capabilities can engender a sustainable competitive advantage^15,16^. It explains how DTA could potentially improve tax avoidance by considering digital infrastructure as a resource that encompasses various digital assets, including software, data analytics tools, and computing resources. DTA can thus be seen as a valuable resource that, when leveraged effectively, improve a firm's ability to identify tax optimization opportunities, reduce tax compliance costs, enhance tax planning, and adapt to changing tax landscapes. Moreover, in China, where EU is a significant factor due to evolving regulations and market dynamics, firms with the right environmental resources and capabilities can improve tax avoidance. These firms can leverage their expertise, data analytics capabilities, strategic alliances, innovation efforts, stakeholder relations, and resource allocation to align their environmental initiatives with tax optimization strategies. Therefore, by effectively managing EU, Chinese firms can reduce tax liabilities while advancing their sustainability goals. Based on the discussion above, this research proposes a framework, as shown in Fig. 1. The development of the hypotheses is discussed in the next section.

## Literature review and hypotheses development

### Corporate green behavior (CGB) and corporate tax avoidance (CTA)

As part of their environmental strategy, many businesses have voluntarily invested in environmental projects, reduced their greenhouse gas emissions, and established green performance targets^14^. Concurrently, both developed and developing countries have instituted market-based environmental control measures like emission levies, pollution licenses, and emission reduction incentives to achieve the UN’s SDGs. Alongside these efforts, legal tax avoidance has emerged as a global phenomenon and a main obstacle for corporations and tax collectors^42^. According to a statistic by UNU's World Institute for Development Economics, CTA results in global tax losses of at least $500 billion annually^43^, with the United States and China ranked as the top two countries affected by this issue^44^.

As of January 1, 2015, China has implemented a new Environmental Protection Law that ensures sufficient penalties for environmental protection violations committed by corporations as well as greater power for environmental law enforcement authorities. Environmental rules can be complicated and difficult to understand, prompting individuals and corporations to look for shortcuts or exploit ambiguities in order minimize their tax burden. For example, firms can change their corporate behavior and reduce environmental taxes by minimizing pollution emissions. Given that avoiding taxes is a cost-cutting economic strategy^10^, Ling et al.^10^ concluded that highly regulated urban polluting industries will engage in more CTA activities as a result of environmental regulation. From the perspective of achieving the SDGs, Chinese firms adopt various green practices (e.g., investments in environmental research and development projects, green patent development), which can contribute to building more sustainable and resilient cities, reducing environmental impact, and improving the quality of life for individuals living in urban areas^11^. To further promote sustainable consumption and production, responsible and ethical tax practices are vital, as they maintain balance and support the generation of funds for public services and sustainable initiatives. Therefore, by assessing the association between CGB and CTA in relation to SDG11 and SDG12, we can assess how businesses' environmental and fiscal practices align with the objectives of creating sustainable communities and promoting sustainable consumption and production patterns. Thus, this study proposes that:

#### H1

CGB significantly influences CTA.

### Moderating role of environmental uncertainty (EU)

EU refers to an unpredictable environmental situation that prompts firms to respond to various challenges, including climate change, natural disasters, customer needs, competition, and technological changes^34^. Frank Knight’s pioneering work distinguishes between EU and risk. Risk pertains to situations where probabilities are known or calculated, seen in activities like gambling or established market investments. In risk management, historical data informs choices, facilitating predictable decision-making. Uncertainty, conversely, arises when probabilities are elusive or hard to gauge, such as the unpredictable consequences of climate change. Consequently, EU requires flexible strategies like scenario planning. Knight’s distinction emphasizes that in environments of uncertainty, firms must adapt and be less reliant on precise probabilities, as unlike risk, uncertainty is not amenable to quantitative analysis and management^38–40^. The study of EU can help identify challenges and opportunities related to urban development, climate change, resource management, and consumption patterns^11^. By addressing these uncertainties, stakeholders can develop strategies that contribute to SDGs 11 and 12.

Previous literature suggests that firms should enhance their business strategy^45^, supply chain management^1^, corporate innovation^46^, and operating investment^47^ to cope with the impact of EU. When non-financial firms face high levels of uncertainty in their operating environment, they are likely to allocate more resources to improving their primary operating activities instead of enhancing their environmental performance^48^. This is because extreme uncertainties (e.g., market volatility, rapid technological changes) are often perceived as significant risks to a firm’s core business operations^34,45^, leading firms to prioritize resource allocation to other areas (e.g., investing in research and development, adapting to market shifts, or securing supply chains) to mitigate these risks and ensure the stability and profitability of their primary operations^18,33^. In contrast, environmental initiatives, although crucial from a sustainability and CSR perspective, may be seen as long-term investments that do not provide the same level of short-term financial returns or risk mitigation as investments in core operations^37,38^. As such, environmental performance may not be seen as an immediate priority during times of high uncertainty. In other words, when making resource allocation decisions, firms tend to allocate more resources to areas that directly impact their financial performance and operational stability, often deferring or reducing investments in environmental performance enhancement until uncertainties in their primary operating activities are better managed or resolved^18,38^.

From the preceding literature, it can be inferred that firms use CGB to comply with tax policies and achieve reasonable CTA. Notably, Huang et al.^46^ argued that managers of small firms operating in uncertain environments prefer to avoid tax, as complex environments offer more tax planning opportunities. Under an environment of uncertainty, firms may adopt a short-term mindset towards their investment returns, leading to increased demand for CTA^49^. EU thus strengthens the connection between CGB and CTA by fostering an atmosphere in which firms are under more pressure to uphold their environmental duty while managing their financial commitments. Moreover, stakeholders (e.g., customers, investors, regulators) pay closer attention to a company’s environmental practices amid uncertain economic times. This heightened scrutiny serves as a strong incentive for businesses to adopt CGB as a means to build trust, enhance their reputation, and maintain competitive advantage^45^. By implementing green practices, firms can align themselves with societal expectations^50^ and meet the growing demand for sustainable solutions. Subsequently, integrating CGB with CTA can provide tax incentives and credits for green practices. This interplay between EU, CGB, and CTA highlights the importance of sustainable practices to navigate uncertain economic landscapes while optimizing financial outcomes. Therefore, it is hypothesized that:

#### H2

EU moderates the effect on CGB on CTA.

### Moderating role of digital technology application (DTA)

DTA is described by Trevisan et al.^30^, as “the practice of applying digital technologies to a specific domain, industry, or business process to achieve efficiency, innovation, growth, and improvement." DTA has various benefits for firms. It reduces information access costs and monitors business trends, which improves corporate governance^51,52^ and mitigates potential risks such as financial fraud^53^. DTA also aids firms in pollution and waste reduction, productivity improvement, responsible consumption, and material recycling and reuse^30,47,54^. It further enables supply chain transparency, traceability, and optimization, thereby reducing environmental impacts and promoting sustainable production practices^55^. Moreover, data analytics and AI can be employed to gather and analyze real-time data on energy use, waste management, and greenhouse gas emissions, which can then be used to identify patterns, optimize processes, and minimize waste generation across various industries^56^. By leveraging digitalization such as smart grids, IoT, and data analytics, cities can improve energy efficiency, optimize resource management, and enhance infrastructure systems^57,58^. For example, smart city solutions can enable efficient transportation systems, optimize waste management processes, and enhance urban planning to create livable and sustainable cities^59^.

Additionally, through digital platforms and tools, consumers can access information about product sustainability, make informed purchasing decisions, and participate in collaborative consumption models. In this manner, DTA can support citizen engagement, facilitating participatory decision-making processes and empowering communities to contribute to sustainable urban development. Overall, the examination of DTA in the context of SDG 11 and SDG 12 has the potential to facilitate the development of sustainable urban areas and communities, as well as encourage sustainable patterns of consumption and production. By harnessing the power of DTA, cities can become smarter and more efficient, while individuals and businesses can make informed choices that reduce environmental impacts and promote sustainable practices.

This study posits that DTA strengthens the connection between CGB and CTA by providing cutting-edge tools and programs that let Chinese firms integrate their environmental and fiscal responsibilities. Businesses can assess their carbon footprint, manage their environmental performance, and implement sustainability projects more successfully through DTA^58^. Digital platforms can also promote accountability and transparency, enabling stakeholders to evaluate a company's environmental practices. These functions enable firms to make informed decisions and embrace their CGB. In addition, by integrating DTA with tax reporting systems, businesses can demonstrate their commitment to environmental responsibility, leading to potential tax benefits and incentives for implementing CGB. This synergy between DTA, CGB, and CTA strengthens the connection between environmental sustainability and fiscal responsibility. Accordingly, this study predicts that:

#### H3

DTA moderates the effect of CGB on CTA.

## Methods

### Research sample

The sample for this study comprised A-share listed companies from Shanghai and Shenzhen, covering the period from 2015 to 2020. Shanghai and Shenzhen were intentionally chosen for this study as they are renowned as China's financial centers. China's listed firms have increasingly embraced CGB in response to environmental challenges and government initiatives. These firms have implemented various measures to reduce their carbon footprint and adopt environmentally responsible practices. For instance, they have improved energy efficiency, utilized renewable energy sources, and implemented waste management systems^60^. By implementing sustainable practices, listed firms contribute to China's environmental goals, attract responsible investment, and enhance their reputation.

The study period from 2015 to 2020 was chosen because it marked a pivotal period of transformation in sustainability practices and policies in China, characterized by significant shifts in environmental priorities, commitments to international sustainability goals, and rapid growth in renewable energy^1,6,7^. First, the full implementation of the country's Environmental Protection Law in 2015 increased environmental regulation costs and environmental governance pressure for corporations. Second, 2020 was the year when the COVID-19 pandemic had just begun, with February to the end of March seeing the entire mainland under quarantine. In the fourth quarter of 2020, China's economy had a small rebound following COVID-19 remission. Since the green behavior of firms is typically a long-term feedback activity, these developments make the 2015–2020 period a crucial and relevant timeframe for conducting sustainability-related studies in China^36^.

The data for the current research was sourced from the reputable CSMAR and EPS databases, as well as from public information disclosed on firms' official websites. Steps taken to check the reliability and accuracy of the data included excluding financial firms from the analysis and carefully identifying and eliminating any missing data and outliers. As a result, the final sample consisted of 467 corporate entities, yielding a robust dataset with a total of 2,802 observations.

### Measurement of variables

#### Dependent variable (DV)

CTA was the dependent variable of this study. Following the studies of Desai and Dharmapala^42^ and Xu et al.^61^, this study used the gap between nominal and effective income tax rates to measure tax avoidance. If the disparity is large, then a substantial amount of taxes was likely avoided.

#### Independent variable (IV)

CBG was considered the independent variable of this study. To measure CGB more comprehensively, this study referred to past literature^62,63^ and adopted three criteria (see Table 1) to reflect a firm’s CGB: green production (GP), circular economy (CE), and green management (GM). In this study, GP denotes the type of energy a Chinese corporation saves during production; CE is based on China's CE indicator system and also refers to a study by Yuan and Pan^52^ to determine whether listed corporations use CE technologies; and GM denotes whether corporations take environmental considerations into account during regular business operations.

**Table 1:** Measurements for corporate green behavior (CGB).

Dimensions	Indicator name
Green production (GP)	Types of energy savings in production: 1. Solar 2. Biomass 3. Hydrogen 4. Wind 5.Ocean 6. Geothermal energy 7. Water Note: 0 is nothing, 1 is company has 1 out of 7, 2 is 2 out of 7, 3 is 3 out of 7, and so on
Circular economy (CE)	Whether it adopts the circular economy Note: 0 is does not adopt the circular economy; 1 is adopts the circular economy
Green management (GM)	1. Whether it has ISO 14001 certification; 2. Whether it adopts a green office; 3. Whether the environmental protection investment amount is disclosed in CSR reports; 4. Whether it has an idea or vision of being responsible for the environment; Note: 0 is nothing, 1 is company has 1 out of 4, 2 is 2 out of 4, and so on

### Moderating variables

The two moderators used in the present study are EU and DTA. EU has been defined in terms of its inherent changeability. The dynamic nature of an organization's external environment is reflected in the degree of uncertainty with which it must plan for the future, which can be approximated by looking at its sales and profit fluctuations^10^. The standard deviation of irregular sales income over the five-year study period was calculated using companies’ operational income data from the same period. Then, the value was revised to account for prevalent industry practices, and the revised figure served as the basis for measuring EU. The measurements and formula employed were based on the research of Ghosh and Olsen^33^ and Purnomo and Eriandani^35^. The formula is shown below:

$${\text{Sale}} = \varphi 0 + \varphi 1{\text{Year}} + \varepsilon$$

where Sale represents operating income and Year represents the annual variable.

DTA is difficult to measure using a single financial indicator^52^. Nevertheless, the extent to which a firm attaches importance to a particular strategic orientation can be reflected by the frequency with which keywords related to that strategy appear in its annual report^52,64^ Therefore, based on DTA measurement standards in past research, DTA was calculated by the percentage of annual reports containing relevant digitalization keywords^65,66^. As digital technology has various types, this study obtained annual data for the 2015–2020 period from firms’ annual reports. The specific measurement of keyword frequency in annual reports was based on the calculation methods in the works of Yuan and Pan^52^ and Zhuo and Chen^66^. The Python software was employed for this purpose, as the Jieba module in Python software can automatically separate text content, extract keywords, and count word frequency. Table 2 lists the DTA keywords^52,66^.

**Table 2:** Description of DTA keywords.

DTA (keywords)	Data management, data mining, data networks, data platforms, data centres, data science, digital control, digital technology, digital communication, digital networks, digital intelligence, digital terminals, digital communication, digital networks, digital intelligence, digital terminals, digital marketing, digitation, big data, cloud computing, cloud IT, cloud ecology, cloud services, cloud platforms, blockchain, Internet of Things, machine learning

### Control variables

This study incorporated control variables based on the literature on environmental regulation and CTA, as previously discussed in Zhang et al.^67,68^. The control variables under consideration in this study were as follows: (1) Size, which refers to the size of the firm and is measured as the logarithm of the number of employees; (2) Lev, which refers to the level of leverage and is measured as the ratio of total liabilities to total assets; and (3) Indep, which refers to the presence of independent directors on the board and is measured as the number of independent directors divided by the total number of directors. The aforementioned variables are regarded as factors that exert an influence on the valuation of a company.

### Model construction

The present investigation established an empirical framework that builds on the theoretical constructs expounded in prior studies by Ong et al.^69^, Ren et al.^53^, Li and Ramanathan^63^, and Omonijo and Yunsheng^70^. By extending and applying these studies’ models to the Chinese setting, this research expands the literature on sustainable communities.

The regression models shown below were formulated in this study.

1 $${\text{CTA}}={\upvarepsilon +\upbeta }_{1}{\text{GP}}+{\upbeta }_{2}{\text{CE}}+{\upbeta }_{3}{\text{GM}}+{\text{Control}}+{\text{Constant}}$$

2a $${\text{CTA}}={\upvarepsilon +\upbeta }_{1}{\text{GP}}+{\upbeta }_{2}{\text{CE}}+{\upbeta }_{3}{\text{GM}}+{\upbeta }_{4}{\text{EU}}+{\upbeta }_{5}{\text{GP}}*{\text{EU}}+{\text{Control}}+{\text{Constant}}$$

2b $${\text{CTA}}={\upvarepsilon +\upbeta }_{1}{\text{GP}}+{\upbeta }_{2}{\text{CE}}+{\upbeta }_{3}{\text{GM}}+{\upbeta }_{4}{\text{EU}}+{\upbeta }_{5}{\text{CE}}*{\text{EU}}+{\text{Control}}+{\text{Constant}}$$

2c $${\text{CTA}}={\upvarepsilon +\upbeta }_{1}{\text{GP}}+{\upbeta }_{2}{\text{CE}}+{\upbeta }_{3}{\text{GM}}+{\upbeta }_{4}{\text{EU}}+{\upbeta }_{5}{\text{GM}}*{\text{EU}}+{\text{Control}}+{\text{Constant}}$$

3a $${\text{CTA}}={\upvarepsilon +\upbeta }_{1}{\text{GP}}+{\upbeta }_{2}{\text{CE}}+{\upbeta }_{3}{\text{GM}}+{\upbeta }_{4}{\text{EU}}+{\upbeta }_{5}{\text{GP}}*{\text{DTA}}+{\text{Control}}+{\text{Constant}}$$

3b $${\text{CTA}}={\upvarepsilon +\upbeta }_{1}{\text{GP}}+{\upbeta }_{2}{\text{CE}}+{\upbeta }_{3}{\text{GM}}+{\upbeta }_{4}{\text{EU}}+{\upbeta }_{5}{\text{CE}}*{\text{DTA}}+{\text{Control}}+{\text{Constant}}$$

3c $$C{\text{TA}}={\upvarepsilon +\upbeta }_{1}{\text{GP}}+{\upbeta }_{2}{\text{CE}}+{\upbeta }_{3}{\text{GM}}+{\upbeta }_{4}{\text{EU}}+{\upbeta }_{5}{\text{GM}}*{\text{DTA}}+\mathrm{Control }+{\text{Constant}}$$

In the equations above, the intercept term is represented by a Constant, Ɛ is the random perturbation term, and β is the regression coefficient for each explanatory variable. In addition, the set of control variables is presented in the equations. Model (1) tests the direct relationship between CGB (i.e. GP, CE, and GM) and CTA. Based on the direct relationship in Model (1), Models (2a), (2b), and (2c) include the moderating variable EU to examine its influence on the relationship between CGB and CTA. Similarly, Models (3a), (3b), and (3c) add DTA as the moderating variable in the relationship between CGB and CTA.

## Results

### Descriptive statistics

The descriptive statistics results (Table 3) illustrate significant variations in CTA among corporations, with an average value of − 0.004, the lowest value being − 0.5041, and the highest value reaching 0.238. In the case of EU, the average is 21.178, with a maximum of 28.718 and a minimum of 7.125, indicating that the level of EU shows relatively minor variation among Chinese firms. GP, CE, and GM were represented by dummy variables in this analysis. The mean of EU is 21.178, with a standard deviation of 1.29, signifying an overall acceptable level of EU among listed firms. Overall, disparities exist in EU and DTA among Chinese listed firms.

**Table 3:** Descriptive statistics.

Variable	Obs	Mean	Std. dev	Min	Max
CTA	2802	− 0.004	0.109	− 0.5041	0.238
Size	2802	22.200	1.29	19.525	26.395
Lev	2802	0.418	0.205	0.052	0.925
Indep	2802	0.377	0.054	0.300	0.600
EU	2802	21.178	1.559	7.125	28.718
DTA	2802	1.978	8.930	0	296
GP	2802	0.020	0.311	0	7
CE	2802	0.374	0.484	0	1
GM	2802	0.105	0.573	0	4

### Correlation matrix

Tables 4 and 5 display the correlation matrices for the research variables, emphasizing the relationship between CGB and its dimensions as well as its relationship with the other variables. In this study, * indicates significance at p < 0.05. Similarly, ** and *** indicate that the results are significant at p < 0.01 and p < 0.001, respectively.

**Table 4:** Correlation matrix.

	CTA	GP	CE	GM	EU	Size	FirmAge	Indep
CTA	1.000							
GP	0.043**	1.000						
CE	− 0.017**	− 0.0140	1.000					
GM	− 0.437***	0.131***	0.046***	1				
EU	0.00200	0.0240	0.409***	0.030***	1			
Size	− 0.040***	0.0240	0.130***	0.169***	0.165***	1.000		
Lev	− 0.163***	0.0168	0.060***	0.032***	0.079***	0.507***	1.000	
Indep	− 0.014*	− 0.00500	− 0.00900	0.00100	0.00600	− 0.018**	− 0.007	1.000

**Table 5:** Correlation matrix.

	CTA	GP	CE	GM	EU	Size	FirmAge	Indep
CTA	1.000							
GP	0.043**	1.000						
CE	− 0.017**	− 0.0140	1.000					
GM	− 0.437***	0.131***	0.046***	1				
DTA	0.042***	0.168***	− 0.019***	0.00900	1			
Size	− 0.040***	0.0240	0.130***	0.169***	− 0.00900	1.000		
Lev	− 0.163***	0.0240	0.060***	0.032***	− 0.051***	0.507***	1.000	
Indep	− 0.014*	− 0.00500	− 0.00900	0.00100	0.021***	− 0.018**	− 0.007	1.000

### Collinearity diagnosis

The results in Table 6 show that the mean of the Variance Inflation Factor (VIF) is 1.229, which is more than, yet close to, 1.0. In the presence of multicollinearity, regression estimates are unstable and have high standard errors. Thus, the results of Table 6 show that no serious multicollinearity exists in this study, which supports the findings’ reliability.

**Table 6:** Collinearity diagnosis.

Variable	VIF	1/VIF
GP	1.010	0.994
CE	1.22	0.821
GM	1.05	0.956
EU	1.26	0.794
DTA	1.04	0.96
Size	1.650	0.607
Lev	1.580	0.632
Indep	1.02	0.994
Mean VIF	1.229	

### Regression result

Table 7 presents the regression outcomes of the panel model, with Model 1 exhibiting the findings of the direct association. The results of Models 2a, 2b, and 2c report on EU’s moderating role in the association between CGB and CTA. To begin, Model 1 reveals that CGB has a statistically significant effect on CTA. Specifically, GP has a direct positive influence on CTA, while the adoption of CE and GM by firms reduces their tax avoidance activities. These findings support the notion that during the 2015–2020 period, the adoption of CGB by Chinese firms had a significant impact on CTA.

**Table 7:** Regression results of models 1 and 2.

	Model 1	Model 2a	Model 2b	Model 2c
	CTA	CTA	CTA	CTA
GP	0.0179*** (4.37)	0.0182*** (4.27)	0.0190*** (4.54)	0.0188*** (4.48)
CE	− 0.00417** (− 2.64)	− 0.00616*** (− 3.52)	− 0.0113*** (− 21.65)	− 0.00603*** (− 3.47)
GM	− 0.0106*** (− 20.67)	− 0.0111*** (− 21.39)	− 0.00567** (− 3.25)	− 0.0114*** (− 21.39)
EU		2.87e−14 (0.66)	3.48e−13*** (3.77)	− 5.46e−14 (− 1.01)
EU*CGB		4.97e−14 (0.89)	− 9.15e−14*** (− 3.32)	1.76e−14* (2.47)
Size	0.0104*** (5.81)	0.0102*** (5.53)	0.00991*** (5.40)	0.0100*** (5.46)
Lev	− 0.0953*** (− 7.51)	− 0.0972*** (− 7.61)	− 0.0952*** (− 7.46)	− 0.0962*** (− 7.54)
Indep	0.0210 (0.60)	0.0302 (0.86)	0.0343 (0.98)	0.0325 (0.93)

In Table 7, Model 2a reveals that EU does not significantly moderate the relationship between GP and CTA, suggesting that the level of EU does not affect GP’s influence on CTA among Chinese listed firms. On the other hand, Models 2b and 2c indicate that the moderating role of EU in the impacts of CE and GM on CTA are significant at the 1% and 10% confidence levels, respectively. The interaction term of EU and CE exhibits a negative value of -3.32, while the coefficient of the interaction between EU and GM is 2.47.

The panel regression results for Model 3 are displayed in Table 8. The interaction terms of Models 3a and 3c are significant, indicating that DTA effectively weakens tax avoidance activities resulting from higher GP and lower GM in Chinese listed firms. However, the interaction term of DTA and CE in Model 3b is not significant. This result infers that DTA does not influence the negative relationship between CE and CTA in Chinese listed firms.

**Table 8:** Regression results of model 3.

	Model 3a	Model 3b	Model 3c
	CTA	CTA	CTA
GP	0.0174*** (4.21)	0.0148*** (3.57)	0.0177*** (4.06)
CE	− 0.00436** (− 2.77)	− 0.00451** (− 2.72)	− 0.0107*** (− 20.83)
GM	− 0.00990*** (− 17.92)	− 0.0107*** (− 20.84)	− 0.00377* (− 2.40)
DTA	0.000943*** (3.38)	0.000837*** (3.60)	0.00158*** (3.93)
DTA*CGB	− 0.000142*** (− 3.38)	0.000504 (1.42)	− 0.000986* (− 2.03)
Size	0.0104*** (5.85)	0.0104*** (5.84)	0.0103*** (5.78)
Lev	− 0.0971*** (− 7.65)	− 0.0921*** (− 7.27)	− 0.0924*** (− 7.30)
Indep	0.0201 (0.58)	0.0197 (0.57)	0.0179 (0.52)
_cons	− 0.153*** (− 3.88)	− 0.151*** (− 3.84)	− 0.149*** (− 3.80)
N	2802	2802	2802
r^2^	0.168	0.149	0.148

### Robustness analysis

To test the robustness of the benchmark regression results, the measurement of the dependent variable CTA was changed to accounting for tax differences (ATD) to portray the extent of CTA in a business^42^. According to Desai and Dharmapala^42^, ATD equals pre-tax accounting profit (after deducting taxable income) divided by total assets at the end of the period. The taxable income can be calculated by dividing the current income tax expense by the nominal income tax rate^71^. According to Jiang et al.^72^, there is a positive correlation between a company's value and the variance between its accounting profit and taxable income. This relationship increases the probability of the company engaging in tax avoidance practices. The robustness test results are consistent with the regression results, as demonstrated in Tables 9 and 10.

**Table 9:** Robustness test results of model 2.

	Model 2b	Model 2c
	ATD	ATD
GP	0.00163 (1.72)	0.00169 (1.78)
CE	− 0.00152*** (− 3.86)	− 0.00208*** (− 17.67)
GM	− 0.00212*** (− 17.61)	− 0.00147*** (− 3.71)
EU	− 2.05e−14 (− 1.67)	6.59e−14** (3.15)
EU*CGB	4.43e−15** (2.75)	− 1.84e−14** (− 2.94)
_cons	0.0142*** (15.28)	0.0139*** (15.16)
N	2792	2792

**Table 10:** Robustness test results of model 3.

	Model 3a	Model 3c
	ATD	ATD
GP	0.00224* (2.28)	0.00116 (1.22)
CE	− 0.00140*** (− 3.94)	− 0.00134*** (− 3.74)
GM	− 0.00190*** (− 16.48)	− 0.00195*** (− **16.13)**
DTA	0.000391*** (4.29)	0.000380*** (4.42)
DTA*CGB	− 0.000330** (− 3.00)	− 0.0000316** (− 2.75)
_cons	− 0.0277** (− 3.11)	0.0128*** (13.79)
N	2832	2832

Significant values are in bold.

**Figure 2:**
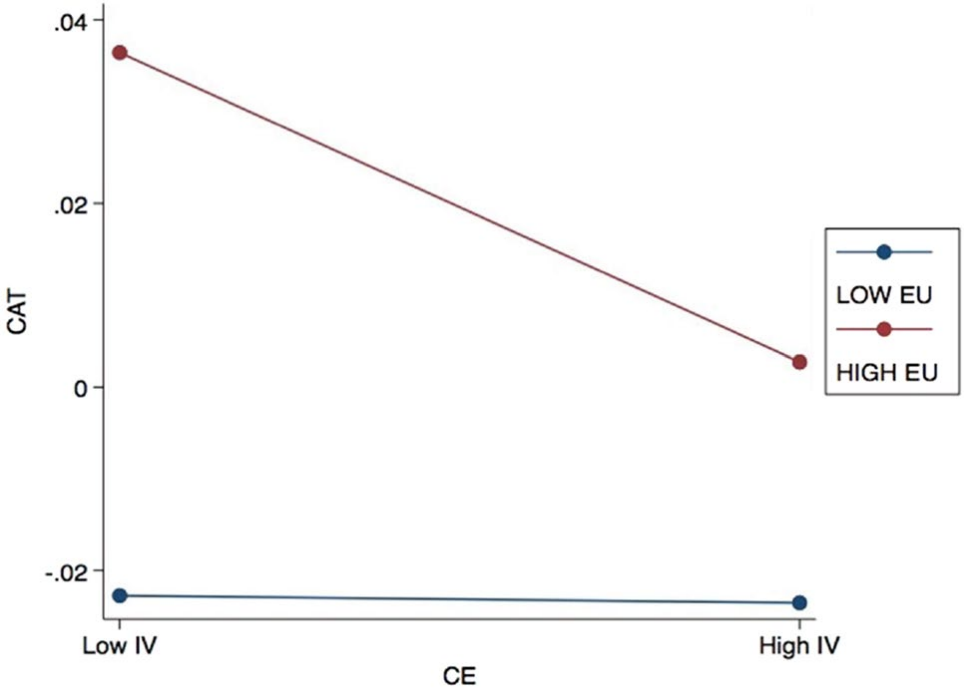
Effects of EU on the relationship between CE and CTA.

**Figure 3:**
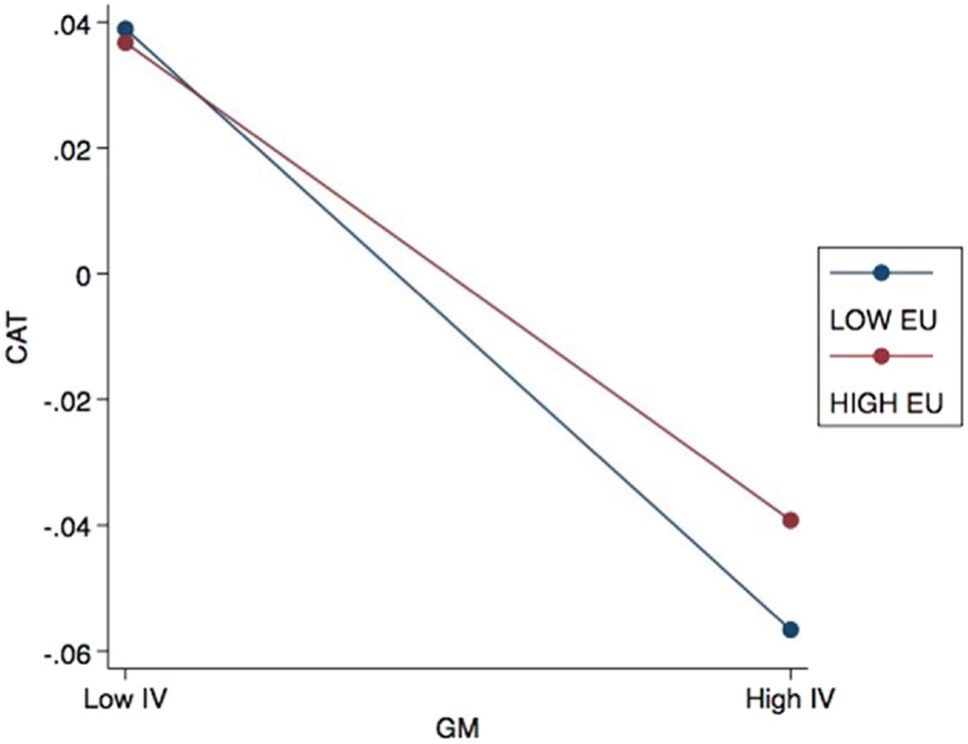
Effects of EU on the relationship between GM and CTA.

This study also conducted additional analyses to check the robustness of the moderation results. The primary model outputs demonstrate statistical significance with minimal deviations at the designated significance level, consistent with the original data. To visually represent the moderating effects under examination, the present investigation employed the Aiken and West^73^ method, as illustrated in Figs. 2 and 3. The diagrams illustrate the below-average (1 standard deviation below the mean) and above-average (1 standard deviation above the mean) impacts of CGB on CTA. Following the theoretical framework tested in Model 2b, Fig. 2 illustrates that higher EU can intensify the adverse effects of CE on CTA. Figure 3, on the other hand, demonstrates that a low level of EU offsets the impact of GM on CTA. These findings suggest that EU involvement in the relationship between CGB and CTA yields mixed results, representing a noteworthy discovery.

**Figure 4:**
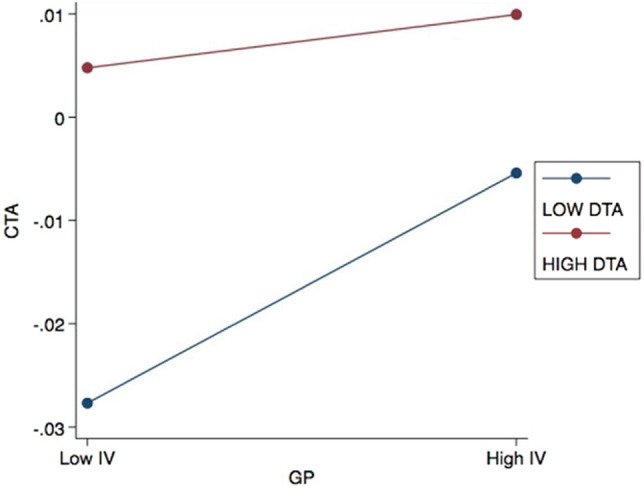
Effects of DTA on the relationship between GP and CTA.

**Figure 5:**
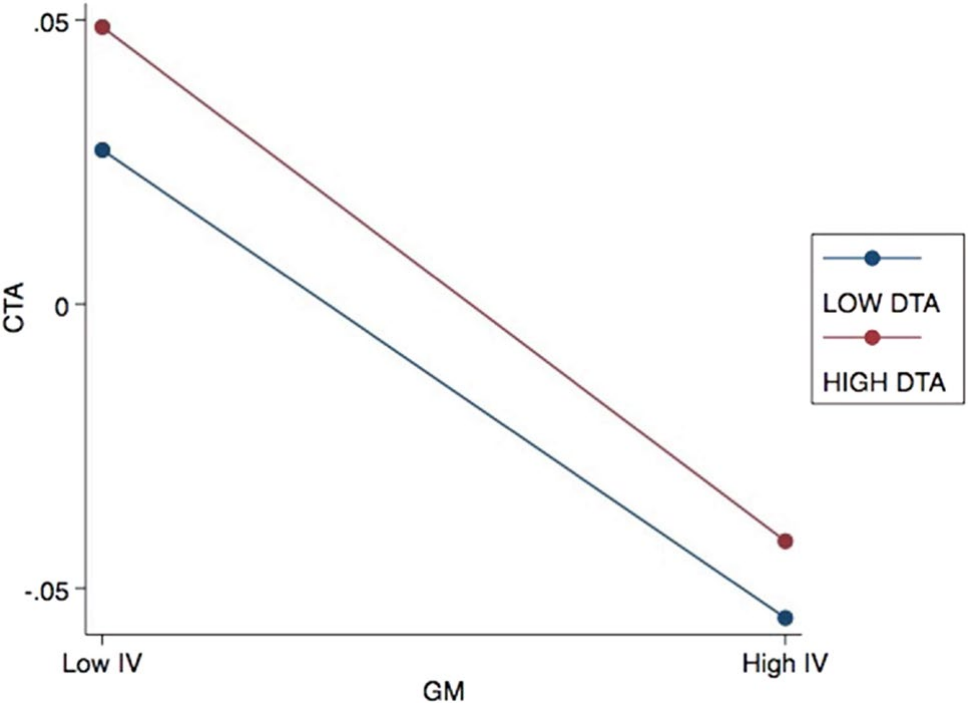
Effects of DTA on the RELATIONSHIP between GM and CTA.

Figures 4 and 5 highlight the moderating role of DTA in the interaction between CGB and CTA. The empirical results indicate that over the 2015–2020 period, the use of digital technologies by listed firms in mainland China weakens the positive impact of GP on their tax avoidance. This implies that these businesses are focusing less on energy-saving for GP due to the potential impact of digital transformation on their business models^32,37^. Consequently, the tax incentives for green products may be reduced for these firms^35,41^. Moreover, DTA enhances the adverse impact of GM on CTA. Firms that use GM and increase their deployment of digital technologies are less likely to avoid taxes, suggesting that mainland Chinese listed firms are leveraging digital transformation technologies to enhance their environmental stewardship even though it hampers their CTA. This aligns with government policy guidelines and indicates that these businesses are increasingly prioritizing environmental protection and sustainable operations to achieve sustainable community goals^32^. Therefore, the statistical outcomes confirm that the use of DTA by firms can have a detrimental effect on the impact of CGB on CTA.

## Discussion

Businesses in China are increasingly recognizing the importance of aligning with environmental practices and the potential of sustainable communities. According to institutional theory, organizations conform to institutional pressures to gain legitimacy, ensure their value creation, and remain relevant. By proactively participating in CGB, businesses demonstrate their commitment to sustainable development and contribute to the achievement of SDG 11 and SDG 12. In parallel, the Chinese government has made notable efforts and policies to promote sustainable practices, prompting corporations to respond accordingly.

The findings of this analysis provide support for the research hypotheses related to the impact of CGB on CTA, as well as the moderating effects of EU and DTA. Hypothesis 1, which posits the significant impact of CGB on CTA, is confirmed by the results. This finding is supported by the stakeholder theory, which proposes that firms can enhance their tax efficiency by adopting CGBs that align with the desires and expectations of diverse stakeholders. The Chinese government is a significant stakeholder with a keen interest in environmental sustainability and tax collection^37,38^. By engaging in CGB, such as reducing carbon emissions and adopting clean technologies, firms align with government policies while simultaneously avoiding the financial penalties associated with regulatory violations. They can also access tax incentives, draw in investors and customers, foster positive community relations, and establish a more streamlined supply chain^34,45^. These collective efforts contribute to improved financial performance, ultimately leading to enhanced tax efficiency.

The building of sustainable communities often requires the collaboration and support of a wide range of stakeholders^33,37,40^, including corporate businesses. Firms play a key role in this regard by taking into account and responding to the interests and aspirations of community residents, governments, environmental organizations, and other stakeholders, ultimately ensuring sustainable community development and promote shared prosperity^37,38^. Correspondingly, the relationship between CGB and CTA supports sustainable communities through its interconnected effects on community expectations, stakeholder engagement, resource efficiency, and resilience. Firms that align with the sustainability goals and values of their communities through CGB practices are more likely to foster positive relationships, contribute to local sustainability efforts, and reduce the temptation to engage in aggressive tax avoidance, thereby creating a mutually beneficial relationship between businesses and their communities.

From an institutional theory perspective, the positive relationship between CGB and CTA can be attributed to the institutional pressures (e.g., regulatory pressures, investor expectations, and media attention) firms face to conform to environmental expectations and gain legitimacy. Chinese firms, in particular, are under pressure from the government and public opinion following the full implementation of the Environmental Protection Law since 2015. Additionally, firms encounter a challenging external environment due to the development goals of sustainable communities^37,40^. By aligning with these expectations, firms can access tax incentives, enhance their reputation, and ensure their long-term survival in an evolving institutional landscape. Therefore, it is evident that engaging in environmentally responsible practices can positively influence a firm's tax avoidance strategies. However, it is important to note that some firms may engage in “greenwashing,” whereby they falsely portray themselves as environmentally friendly to gain consumer trust and benefit from tax incentives associated with being a green business. This practice can lead to government tax incentives being misused or manipulated for financial gain if firms exaggerate their green efforts without a genuine commitment to sustainability or substantial green initiatives^50^.

While some studies have demonstrated a statistically significant relationship between CGB and CTA, others have reported otherwise. This indicates that the CGB-CTA link is complex and context-dependent. Accordingly, when examining the moderating roles of EU and DTA on this relationship between CGB and CTA, this study revealed mixed results. The analysis for Hypothesis 2 reveals that EU acts as a moderator in the negative relationship between CE and CTA (Model 2a). Additionally, Model 2c demonstrates that EU moderates the negative relationship between GM and CTA. These findings suggest that the level of EU influences the extent to which CE practices and GM impact Chinese firms’ CTA behaviors.

Overall, EU plays a moderating role in the relationship between CGB and CTA. According to institutional theory, organizations adhere to institutional norms and expectations to establish legitimacy and secure their long-term viability. In the case of CGB and CTA, organizations may face different pressures and constraints depending on the level of EU they encounter, leading to variations in CTA behaviors. Firms operating in high-uncertainty environments may prioritize CGB to mitigate risks associated with environmental compliance and stakeholder pressures. These firms recognize the importance of sustainability in maintaining their reputation, attracting customers, and retaining stakeholder support. In such cases, the focus on CGB may take precedence over aggressive tax planning, as the potential negative consequences of being perceived as environmentally irresponsible outweigh the benefits of tax avoidance. On the other hand, firms operating in lower uncertainty environments may have more clarity and stability in terms of environmental regulations and stakeholder expectations. These firms may be able to pursue both CGB and CTA simultaneously, as they can navigate the regulatory landscape more effectively and identify opportunities for tax optimization within legal boundaries.

Additionally, sustainable communities often experience environmental uncertainties related to climate change, resource availability, and regulatory changes^33,38^. Firms operating in such environments may assess these risks^31^ and recognize that embracing CGB practices can help them adapt to unpredictable environmental conditions. This strategic adaptation can lead to a reduction in CTA as businesses prioritize long-term sustainability over short-term tax savings. Both SDG 11 and SDG 12 require businesses to adopt environmentally responsible practices and contribute to a sustainable future. In line with the stakeholder theory, the results of this study reveal the efforts made by listed firms in mainland China in responding to and working towards these goals, with firms prioritizing resource efficiency and CE principles to adopt green behaviors that are environmentally responsible^32^. However, it should be acknowledged that the level of EU within a given context can shape the relationship between CGB and CTA in achieving these goals.

Regarding Hypothesis 3, the results indicate that DTA moderates the relationship between GP and CTA (Model 3a). Similarly, Model 3c provides evidence for DTA’s moderating effect on the relationship between GM and CTA. This suggests that the use of digital technologies influences the effectiveness of GP and GM strategies in affecting tax avoidance. Overall, the empirical findings demonstrate that DTA reduces Chinese firms’ tax avoidance through CGB,. The application of digital technologies to strengthen the connection between CGB and CTA is consistent with the RBT. According to this theory, a firm's unique resources and capabilities have an impact on its competitive advantage and long-term performance. Integrating DTA with tax reporting systems creates synergies between environmental sustainability and fiscal responsibility, in line with the RBT's notion of resource complementarity, where the combination of different resources amplifies their benefits. By utilizing digital tools to optimize green behavior, firms can potentially unlock tax benefits and incentives, leveraging their digital and environmental resources to achieve financial advantages^54^. This highlights the significance of DTA as a valuable resource that can be leveraged to enhance both a firm's environmental practices and financial outcomes. Indeed, Di Vaio et al.^41^ emphasized the significance of utilizing distinct resources and capabilities, specifically DTA, to establish competitive advantages and enhance both environmental and financial outcomes.

This finding could also be attributed to the cost reduction enabled by DTA, allowing firms to accurately analyze consumer requirements in response to market changes and promptly conduct research and development^52,64,70^. Furthermore, DTA can help firms optimize resource use within sustainable communities. For instance, smart building systems can reduce energy consumption, aligning with CGB objectives. These efficiency gains can positively impact a company's financial performance and potentially reduce the need for aggressive tax avoidance^37,38^. Resource-efficient practices made possible by digital technology, such as reducing water and energy consumption, contribute to sustainability goals and demonstrate a commitment to responsible resource management^18,19^.

This study addresses the complex interactions between firms’ environmental concerns and economic outcomes within a sustainability framework that includes local communities. Despite limited attention on this topic in the current body of literature, the findings provide empirical evidence on the significant association between CGB and CTA, as well as the moderating impacts of EU and DTA in this relationship. Specifically, the adoption of GP by Chinese listed firms promotes CTA, while CE and GM reduce CTA. Firms’ GP adheres to the latest requirements of China's environmental protection laws and therefore facilitates CTA activities. In contrast, CE and GM do not directly reflect environmental protection laws, despite being in line with other energy-saving policies^74^; thus, they reduce CTA. Prior research^41,75^ has likewise argued that the link between CE and sustainable business strategies remains ambiguous. Further, while some firms may use the concept of CE to obtain tax incentives and gain other benefits from the government^76^, such as by falsely advertising or labelling substandard products as recyclable, higher EU can exacerbate the negative impact of CE adoption on CTA. On the other hand, EU weakens the negative impact of GM on CTA. Past research has highlighted that coercive policies can be detrimental and lead to reduced profitability or increased spending on environmental responsibility, which may be managed through higher levels of CTA^77^.

In the pursuit of SDG 12, developing nations are encouraged to undertake measures such as tax restructuring and the gradual elimination of harmful subsidies, where applicable. These actions should be implemented with careful consideration to mitigate any potential negative consequences on overall development while simultaneously safeguarding the welfare of impoverished individuals and affected communities. In China, the growing emphasis on environmental consciousness has put pressure on both businesses and government agencies to adopt environmentally responsible behavior and sustainable practices. Consequently, firms are increasingly embracing CGB activities to lessen their environmental impact, including using energy-saving techniques, reducing emissions, and fostering sustainable resource management^50^. Government agencies are also enacting laws and guidelines that promote and enforce environmentally friendly behavior^58^. This pressure and the resulting actions taken by businesses and government agencies reflect the urgent need to address environmental challenges.

## Implications

Studying the interrelationships among CGB, CTA, EU, and DTA within the framework of SDG 11 and SDG 12 has significant academic implications. Notably, it addresses a critical literature gap by integrating these concepts, shedding light on the complex dynamics and trade-offs between environmental sustainability and financial strategies in sustainable development. Investigating the moderating role of EU provides insights into adapting green behavior and tax strategies in unpredictable environmental landscapes, whereas analyzing the moderating effect of DTA highlights technology's transformative potential in aligning CGB and CTA with the SDGs. Ultimately, this research extends academic knowledge about the interplay between environmental sustainability, financial strategies, and the achievement of SDGs, fostering a holistic understanding of sustainable development.

From a practical perspective, understanding the linkage between CGB, CTA, EU, and DTA informs strategic decision-making. This insight aids organizations in developing holistic sustainability strategies that merge environmental and financial aspects, consistent with stakeholder theory. First, businesses can enhance CGB efforts, optimize resource management, and uncover tax advantages using digital technology, leading to operational efficiency, eco-friendliness, a better reputation, and cost savings. Second, investors can consider CGB and CTA practices when making sustainable investment decisions, aligning with stakeholders' views and long-term wealth creation. This is especially crucial as environmentally conscious customers support businesses with strong CGB and ethical tax practices. Lastly, the research results encourage the strategic adoption of digital technology for improved environmental monitoring, resource tracking, and operational optimization. This, alongside tax considerations, leads to informed decision-making, transparency, and efficiency in achieving CGB goals. Ultimately, these practical implications promote comprehensive sustainability in consumption and production by aligning CGB, CTA, EU, and DTA, thereby contributing to SDG 11 and SDG 12.

## Conclusion

In conclusion, this study not only reaffirms the substantial impact of CGB on CTA, as viewed through the lenses of stakeholder theory, institutional theory and RBT, but also sheds light on the moderating influences of EU and DTA in shaping the dynamics between sustainability practices and tax avoidance^20,62^. Beyond these findings, it underscores a more challenging argument—the imperative for businesses to cultivate CE policies. By demonstrating how EU and the strategic use of digital technology can mitigate the effect of CE and GM on tax avoidance behaviors among Chinese listed firms, this study highlights the evolving landscape of sustainable practices.

Furthermore, the study reiterates the broader significance of sustainable communities in this context. Sustainable communities play a pivotal role in harnessing natural resources efficiently and fostering a resilient economy. Their influence extends beyond business operations, facilitating the transition toward a more environmentally friendly and sustainable future. As businesses and communities continue to collaborate and adapt in the face of environmental uncertainties, the nexus between CGB, CTA, and sustainable communities becomes an even more challenging and vital area of exploration, warranting further research and attention.

## Data Availability

All data generated or analysed during this study are included in this published article (and its Supplementary Information files).

## Abbreviations

CGB: Corporate green behavior
CTA: Corporate tax avoidance
EU: Environmental uncertainty
DTA: Digital technology application
SDGs: Sustainable development goals
RBT: Resource-based theory
UN: United nations
GP: Green production
CE: Circular economy
GM: Green management
